# Adenosine Receptor Functionality and Desensitization Machinery in a Neuronal Cell Model of Angelman Syndrome

**DOI:** 10.3390/jdb14020020

**Published:** 2026-05-02

**Authors:** Martina Contestabile, Jacqueline Fátima Martins de Almeida, Chiara De Cesari, Ilaria Tonazzini, Paolo Giovanni Artini, Simona Daniele

**Affiliations:** 1 Department of Pharmacy, University of Pisa, 56126 Pisa, Italy; m.contestabile@student.unisi.it (M.C.); jacqueline.moraes@farm.unipi.it (J.F.M.d.A.); simona.daniele@unipi.it (S.D.); 2 Istituto Nanoscienze, Consiglio Nazionale delle Ricerche (CNR), @NEST-SNS, Piazza San Silvestro 12, 56127 Pisa, Italy; chiaradecesari@cnr.it (C.D.C.); ilaria.tonazzini@cnr.it (I.T.); 3 Department of Clinical and Experimental Medicine, University of Pisa, 56126 Pisa, Italy

**Keywords:** angelman syndrome, adenosine receptors, signaling pathways, receptor desensitization

## Abstract

Angelman syndrome (AS) is a neurodevelopmental disorder caused by the loss of maternal UBE3A expression, leading to disrupted proteostasis and synaptic dysfunction. Adenosine is a ubiquitous neuromodulator whose G protein-coupled receptors (ARs) regulate neuronal differentiation and neurite outgrowth during development. Here, we investigated AR signaling and their influence on survival–autophagy balance and neuronal morphology in an AS cellular model. Using SH-SY5Y cells with silenced UBE3A, we found that UBE3A loss markedly decreased A1AR, A2BAR, and A3AR protein levels while significantly increasing A2AR expression. Ligand affinity was preserved across genotypes, but A1AR and A2AAR desensitization kinetics were significantly slower in UBE3A-deficient cells. These effects were associated with reduced recruitment of G protein-coupled receptor kinase 2 (GRK2) to the plasma membrane and decreased GRK2–AR association in UBE3A-deficient cells, suggesting a possible contribution of altered GRK2 dynamics to prolonged AR signaling. Functionally, A1AR and A2AR agonists preferentially promoted survival of UBE3A-deficient cells and modulated the MDM2–p53 axis and autophagy markers; A1R stimulation also increased neurite density in UBE3A-deficient cells. Together, these results identify AR-level alterations and defective desensitization machinery in AS neuronal cells and link receptor changes to downstream proteostasis and morphological phenotypes relevant to AS pathophysiology.

## 1. Introduction

Angelman syndrome (AS) is a rare neurodevelopmental disorder caused by loss of function of the maternally inherited UBE3A gene, which encodes an E3 ubiquitin ligase essential for neuronal proteostasis and synaptic function [1]. In neurons, the paternal UBE3A allele is epigenetically silenced by genomic imprinting, and so maternal deletions, mutations, or inactivating defects produce a near-complete loss of neuronal UBE3A and lead to widespread synaptic and circuit dysfunction. Clinically, AS is characterized by severe developmental delay, intellectual disability, absent speech, epilepsy, sleep disturbances, and prominent motor problems, including ataxia and impaired coordination [2,3,4].

Adenosine is a ubiquitous neuromodulator whose G protein-coupled receptors (A1, A2A, A2B, A3 adenosine receptors, ARs) exert subtype-specific control over intracellular signaling cascades that regulate neuronal differentiation and morphological maturation [5]. Beyond their roles in mature synaptic modulation [6,7], ARs act as trophic regulators during development: adenosine and related purines promote neuronal differentiation and neurite outgrowth in multiple models, and AR subtype activation differentially controls intracellular cAMP/PKA signaling and downstream cytoskeletal effectors that drive neuritogenesis [5]. Functionally, A1 and A3 ARs couple predominantly to Gi/o proteins to inhibit adenylyl cyclase, whereas A2A and A2B ARs couple to Gs proteins to stimulate cAMP production; these opposing effects on cAMP/PKA signaling provide a mechanistic basis for how AR subtypes differentially influence cytoskeletal dynamics and neurite extension. Indeed, ARs act as trophic regulators during neuronal development, influencing neuronal differentiation, cytoskeletal remodeling, and neurite outgrowth [8]. Adenosine and related purines promote neurite extension and maturation in several neuronal models, and these effects are mediated through subtype-specific modulation of intracellular cAMP/PKA signaling and downstream cytoskeletal effectors. In particular, A2AAR activation has been shown to enhance neurite elongation and growth-cone dynamics, whereas A1AR signaling can modulate neurite outgrowth in a context-dependent manner, depending on developmental stage and co-existing trophic cues [9,10].

These distinct G-protein couplings underlie the divergent effects of AR subtype activation on downstream pathways controlling survival and autophagy, providing a mechanistic rationale for the cAMP-based assays and subtype-selective pharmacology used in this study [11,12,13].

Dysregulation of AR signaling during critical developmental windows may therefore contribute to the synaptic and circuit-level abnormalities observed in neurodevelopmental disorders, including AS. In this sense, a recent study [5] has shown that A_2A_AR over-activation or altered adenosine tone contributes to synaptic imbalances in the cerebellum and striatum that underlie motor deficits in AS, and that pharmacological A_2A_AR blockade can partially restore synaptic marker levels and improve motor outcomes in AS mice. However, precise cellular mechanisms and determining whether A_2A_AR targeting can modify other AS phenotypes or produce durable benefits require further study [5]. Comprehensive analyses of adenosine signaling across development underscore its dynamic and context-dependent nature in terms of receptor expression and adenosine availability, which shape neuronal excitability, neurotransmitter release, and long-term plasticity [14], thus suggesting that altered adenosine signaling may represent both a mechanistic contributor to NDD pathophysiology and a potential therapeutic target [6,7,14]. Moreover, the temporal control of AR signaling is governed by receptor phosphorylation and trafficking: G protein-coupled receptor kinase 2 (GRK2) phosphorylates activated GPCRs, promoting β-arrestin recruitment, functional desensitization, and receptor internalization, thereby shaping the amplitude and duration of downstream signaling. Given GRK2’s central role in terminating GPCR responses, we examined whether altered GRK2 recruitment or GRK2–AR complex formation contributes to the prolonged AR signaling and altered desensitization kinetics observed in UBE3A-deficient cells [15,16,17,18].

This paper aims to characterize expression, signaling and functional responses of adenosine receptors in Angelman syndrome neuronal models. We investigated in UBE3A-silenced SH-SY5Y neuroblastoma cells, how AR signaling modulates cAMP dynamics, AR receptor trafficking, and GRK2 interactions. We also assess the downstream cross-talk of ARs with autophagy and cell survival processes, with the goal of identifying AR-dependent mechanisms that could be targeted to ameliorate AS-related dysfunctions.

## 2. Materials and Methods

### 2.1. Cell Cultures

For in vitro experiments we used the human neuroblastoma cell line SH-SY5Y. Both wild-type (WT) and UBE3A-silenced (AS model, UBE3A^−^) [19] SH-SY5Y cells were employed; these cell lines were kindly provided by Prof. M. Scheffner (Univ. of Kostanz). Cells were maintained in DMEM/F-12 medium supplemented with 10% fetal bovine serum (FBS) and 1% penicillin/streptomycin. To preserve selection in the UBE3A^−^ population, puromycin (1 µg/mL; P7130, MERCK, Darmstadt, Germany) was added at each medium change. Medium was replaced three times per week, and cultures were passed when they reached roughly 80% confluence, following established protocols.

### 2.2. Cell Pharmacological Treatments

To assess the immediate effects of AR agonists’ stimulation and potential receptor translocation in vitro, SH-SY5Y WT and SH-SY5Y UBE3A-silenced cells were seeded in 100 mm culture dishes and, upon reaching ~90% confluence, treated for 30 min with the A_1_ receptor agonist N6-cyclohexyladenosine (CHA) and the A_2a_ receptor agonist 5′-N-ethylcarboxamidoadenosine (NECA) at 10 nM. Cell pellets were collected and stored at −80 °C until processing for immunoenzymatic assays and Western blot analysis. For experiments addressing autophagy and the proteasome system, cells were exposed to the same agonist treatments for 24 h; pellets were then collected and stored at −80 °C for subsequent Western blot analysis. For autophagic flux analysis, cells were treated with DMSO (control), CHA or NECA (10 nM), bafilomycin A1 alone (200 nM), or CHA/NECA in combination with bafilomycin A1. Bafilomycin A1 was added during the last 4 h of treatment following 20 h of agonist exposure. For cAMP assays, we tested CHA, a selective receptor agonist of A_1_R, at 100 nM (C9901, Sigma Aldrich, St. Louis, MO, USA), NECA, a non-selective agonist receptor of A_2B_AR, at 100 nM (35920-39-9, Merck, Darmstadt, Germany), Bay606583, a selective agonist of A_2B_AR, at 50 nM (60-6583, Cayman Chemical Company, Ann Arbor, MI, USA), and Chloro-IB-MECA, a selective agonist of A_3_AR, at 5 nM (C3601, TCI, Tokyo, Japan). For the vitality assay, cells were treated with AR agonists and antagonists, either alone or in combination. To further assess ARs’ specificity, cells were treated with: A_1_AR agonist CHA (10 nM) in the presence of the selective A_1_ receptor antagonist DPCPX (AMab120396, Prodotti Gianni, Milan, Italy) (20 nM); A_2a_AR agonist CGS21680 (C141, Sigma Aldrich) (30 nM) combined with the A_2A_ receptor antagonist ZM241385 (Z0153, Sigma Aldrich) (20 nM); A_2A_AR agonist NECA (10 nM) in the presence of antagonist ZM241385 (14 nM). Cell proliferation was evaluated 24 and 72 h after treatment.

### 2.3. Cell Metabolic Activity—MTS

MTS assay (CellTiter 96^®^ AQueous One Solution, Promega Fitchburg, WI, USA) was used to assess cellular metabolic activity/viability at 24 h and 72 h after treatment. Because MTS reduction reflects mitochondrial/metabolic activity rather than absolute cell number, results are reported as changes in metabolic activity and interpreted cautiously with respect to proliferation. Absorbance was read at 490 nm using a multimode plate reader. For each plate, raw absorbance values were first corrected by subtracting the mean absorbance of cell-free wells (blank). Corrected values were then normalized to the mean of the corresponding untreated wells (control cells or UBE3A^−^ cells) on the same plate (set to 100%). Data are presented as percentage of control (mean ± SEM). When experiments were performed across multiple plates/days, each plate included its own untreated controls and all values were normalized per plate before pooling. The MTS signal reports mitochondrial reductive capacity and is used here as a proxy for metabolic activity/viability rather than a direct cell-count measure.

SH-SY5Y WT and SH-SY5Y UBE3A-silenced cells were treated with AR agonists at concentrations selected according to preliminary dose–response and viability experiments; drugs’ concentrations were selected to exclude cytotoxic effects. Specifically, cells were exposed to CHA (10 and 100 nM), NECA (10 and 100 nM), BAY 60-6583 (5 and 50 nM), and Cl-IB-MECA (0.5 and 5 nM) for 24 h and 72 h.

To further evaluate AR subtypes’ specificity, additional experiments were performed using combined AR agonists and antagonists treatments. Cells were treated with CHA (10 nM) in the presence of the selective A_1_ receptor antagonist DPCPX (20 nM), with the A_2A_ receptor agonist CGS21680 (30 nM) in combination with the A2B receptor antagonist ZM241385 (14 nM), or with NECA (10 nM) in the presence of ZM241385 (14 nM). MTS assay was measured at 24 h and 72 h following treatment.

### 2.4. Protein Expression by Western Blot Analysis

SH-SY5Y cell pellets were lysed in RIPA buffer (9.1 mM NaH2PO4, 1.7 mM Na2HPO4, 150 mM NaCl, pH 7.4, 0.5% sodium deoxycholate, 1% Nonidet P-40, 0.1% SDS) with protease inhibitors and sonicated (35% amplitude, 3 × 30 s), then incubated for 2 h at 4 °C.

To separate membrane fractions, cells were homogenized on ice using a Potter-Elvehjem homogenizer with ~30 strokes to ensure mechanical disruption, in lysis buffer (10 mM TRIS, pH 7.4, 5 mM EDTA, 5 µg/mL benzamidine, and protease inhibitors cocktail). Cells were then centrifuged at 40,000× *g* for 20 min at 4 °C. The pellet, containing the membrane fraction, was resuspended in the same lysis buffer. Membrane fraction was then processed for Western blot analysis following standard lysate preparation protocols.

After protein quantification, equal amounts (~40 µg) were mixed with Laemmli buffer, and separated on 7.5% stain-free precast SDS-PAGE gels (Bio-Rad, Bio-Rad Laboratories, Hercules, CA, USA, version 2020). After electrophoresis, gels were activated and imaged using the ChemiDoc XRS+ imaging system (Bio-Rad) to visualize total protein per lane according to the manufacturer’s instructions. Proteins were then transferred to PVDF membranes (Bio-Rad, Milan, Italy). Membranes were blocked in 5% milk in TBS-0.1% Tween for 60 min and incubated overnight at 4 °C with primary antibodies diluted in 5% milk TBS-Tween. The following day, membranes were washed (3 × 5 min in TBS-0.1% Tween) and incubated with the appropriate secondary antibodies for 2 h at room temperature on a shaker. Chemiluminescent detection (ECL, Bio-Rad) was used to visualize primary antibody binding. Primary antibodies included: MDM2 (sc-5304, Santa Cruz, Dallas, TX, USA; 1:200), LC3β (sc-271625, Santa Cruz; 1:500), phospho-p53 (sc-101762, Santa Cruz; 1:500), GRK2 (sc-562, Santa Cruz; 1:200), A_1_AR (AAR-006, Alomone labs, Jerusalem, Israel; 1:500), A_2A_AR (PA1-042 Invitrogen, Carlsbad, CA, USA; 1:500), A_2B_AR (PA-77849, Invitrogen; 1:500), A_3_AR (PA5-36350, Invitrogen; 1:500). β-actin (MAB1501, Sigma Aldrich; 1:1000) was used as a control to verify the presence of proteins in the membrane fractions and to ensure comparable protein loading across samples. Densitometric quantification of immunoreactive bands was performed with Image Lab Software (Bio-Rad) and results were expressed as percentage of control (WT = 100%). All blots were acquired using the ChemiDoc XRS+ system (Bio-Rad), and band intensities were normalized to the stain-free total protein signal of the corresponding lane, thereby correcting for potential differences in protein loading and transfer efficiency and avoiding the use of housekeeping proteins. Data were expressed as percentage of control (WT = 100%). Additional densitometric processing was performed using ImageJ (ImageJ-win64).

### 2.5. Measurement of Cyclic AMP Levels in SH-SY5Y Cells

Adenosine receptor subtypes display well-characterized and opposing effects on intracellular cyclic AMP (cAMP) through their canonical G-protein coupling: A1R and A3R primarily couple to Gi/o proteins and inhibit adenylyl cyclase, whereas A2AAR and A2BAR couple to Gs proteins and stimulate adenylyl cyclase [5]. Consequently, changes in intracellular cAMP provide a direct, subtype-relevant functional readout of AR activation and of alterations in receptor coupling or efficacy. In our assays, Gi-mediated responses were measured as the inhibition of forskolin-stimulated cAMP accumulation to increase dynamic range and sensitivity, while Gs-mediated responses were measured as agonist-induced cAMP elevation. To minimize confounding by endogenous adenosine, experiments were performed in the presence of adenosine deaminase (ADA). Dose–response and desensitization kinetics were quantified using within-plate controls and plate-normalized values to ensure comparability across experiments.

Both SH-SY5Y WT and SH-SY5Y UBE3A^−^ cells were plated in 24-well plates, at a density of 4 × 10^4^ cells per well, and cultured for 48 h in complete medium (DMEM/F-12 supplemented with 10% FBS and 1% penicillin/streptomycin). Cells were then rinsed with 1× PBS and pre-incubated for 15 min at 37 °C in serum- and antibiotic-free DMEM/F-12 containing 20 µM Ro-20-1724 (557502, Sigma Aldrich) and adenosine deaminase (ADA, 1:2000 dilution, 10102121001, Roche, Basilea, Switzerland). Subsequently, cells were stimulated for 15 min at 37 °C with increasing concentrations (0.5, 1, 5, 10, 50, 100, and 500 nM) of AR agonists (CHA, NECA, Bay 60-6583 or Cl-IB-MECA). DMSO (0,1%) was used as vehicle control. For Gi-coupled receptor agonists (CHA and Cl-IB-MECA), cells were co-treated with 10 µM forskolin (FK); an additional control (DMSO + 10 µM forskolin) was included. After stimulation, cells were washed and intracellular cAMP levels were quantified using a commercial assay kit (ab138880, Abcam, Cambridge, UK) following the manufacturer’s instructions. The kit fluorescence was read on an EnSight™ multimode plate reader (PerkinElmer, Waltham, MA, USA).

### 2.6. Adenosine Receptor Desensitization Kinetics

Desensitization experiments followed the same cAMP measurement protocol as above (par. 2.6), modified to investigate receptor functionality over time. WT and UBE3A-deficient SH-SY5Y cells were pretreated with agonist (100 nM CHA or NECA) for increasing intervals of varying time (5, 15, 30, 60, and 120 min), washed, and then challenged with a standardized secondary stimulation: they were incubated for 15 min at 37 °C in incomplete medium containing the phosphodiesterase inhibitor Ro-20-1724 (20 µM) and ADA, followed by re-stimulation with agonists (10 nM CHA+ 10 µM FK, 10 NECA). Intracellular cAMP was then measured with the commercial kit (ab138880, Abcam), as above, and cAMP values were interpolated from their respective standard curves, as in [20,21]. The residual cAMP response was expressed as nanomol of cAMP and compared to the 0 min (no pre-treatment) response and fitted to obtain desensitization kinetics (t½).

### 2.7. Lysate Samples Preparation for Immunoenzymatic Assays

Subconfluent WT and UBE3A^−^ SH-SY5Y cultures were rinsed with ice-cold 1× phosphate-buffered saline (PBS), scraped, pelleted by centrifugation, and resuspended in lysis buffer (20 mM Tris-HCl, 137 mM NaCl, 10% glycerol, 1% NONIDET-40, 2 mM EDTA, pH 8) containing 1% protease inhibitor cocktail (Sigma Aldrich, Milan, Italy). Cortical tissue samples (~30 mg) were homogenized in the same lysis buffer and sonicated on ice (30 s, repeated three times). Lysates were clarified by centrifugation at 15,000× *g* for 15 min at 4 °C and the supernatants were collected. Protein concentration was measured and adjusted to load 30 µg of protein per well, as described previously for these cells [21,22].

### 2.8. Adenosine Receptors and GRK2 Complex Formation Under Agonist Stimulation

To evaluate GRK2–adenosine receptor complex formation, we used an in-house immunoenzymatic assay [21,23]. After 30 min of agonists’ stimulation, cell lysates were prepared as described above and approximately 30 µg of protein per sample was used. Microplates were coated overnight at 4 °C with a mouse monoclonal anti-GRK2 antibody (#sc-13143, Santa Cruz, Dallas, TX, USA) diluted 1:100 in 0.1 mg/mL polyornithine. Following a 2 h blocking step at 37 °C with 1% bovine serum albumin (BSA), samples were added and incubated for 1 h at room temperature. Primary and secondary antibodies were diluted in 5% milk in TBS-Tween and incubated for 1–1.5 h at 37 °C. For detection, we used rabbit monoclonal anti-A_2A_-R (#sc-13937, Santa Cruz) or anti-A_1_-R (#sc-28995, Santa Cruz) at 1:300, and an HRP-conjugated anti-rabbit secondary antibody at 1:3000. Plates were washed between steps with PBS containing 0.01% Tween. After TMB development, absorbance was read at 450 nm. Results are expressed as percentages relative to controls, with WT values set to 100%.

### 2.9. Cellular Morphology in SH-SY5Y Cellular Model

Neuronal differentiation was induced with retinoic acid (RA, 10 µM) for 3–4 days prior to morphology and MTS assays. RA differentiation of SH-SY5Y cells is known to promote neurite outgrowth and neuronal marker expression while reducing proliferative capacity (decreased Ki-67 and S-phase labeling), and therefore differentiated cultures are considered largely post-mitotic for the time window used here [24,25].

SH-SY5Y WT and SH-SY5Y UBE3A-silenced cells were seeded at a density of 20,000 cells per well, in 24-well multiwell plates. After 3 days in culture, neuronal differentiation was induced by treatment with retinoic acid (RA, 10 µM) alone, or in combination with adenosine receptor agonists: CHA (10 nM) + RA or NECA (10 nM) + RA. Treatments were incubated for 3 days, after which the culture medium was replaced and cells were re-treated with the same compounds. On day 4, cells were imaged using a Zeiss Axio Observer microscope equipped with an Axiocam 208 color camera (Carl Zeiss, Oberkochen, Germany), equipped with a 20× objective, and digital bright-field images were acquired for subsequent morphometric analysis. Neuronal morphology was assessed by measuring neuritic density and was calculated as the total neurite length divided by the number of neurites (µm), with a minimum length threshold of 10 µm. Morphometric analyses were performed using ImageJ software (ImageJ-win64). In each experiment, for each experimental condition, two independent wells were analyzed, and two representative images were acquired from each well, resulting in a total of four images per condition; each condition was tested (*n* = 2) independently. Quantitative analysis was performed independently on each image, and the resulting values were averaged per condition for statistical analysis. A minimum of 20 cells per image were analyzed.

### 2.10. Statistical Analysis

Statistical analyses were conducted using GraphPad Prism (version 10.0; GraphPad Software, San Diego, CA, USA). Data are shown as mean ± SEM from at least three independent experiments. Comparisons between control (WT) and UBE3A^−^ (AS) groups were made using Student’s *t*-test (unpaired, two-tailed). When experiments included multiple variables (e.g., genotype and treatment), statistical significance was determined using two-way ANOVA followed by Bonferroni’s multiple comparisons test. A *p*-value < 0.05 was considered statistically significant. Significance levels are indicated as: * *p* < 0.05, ** *p* < 0.01, *** *p* < 0.001, **** *p* < 0.0001 versus control.

## 3. Results

### 3.1. Adenosine Receptor Expression

**Figure 1:**
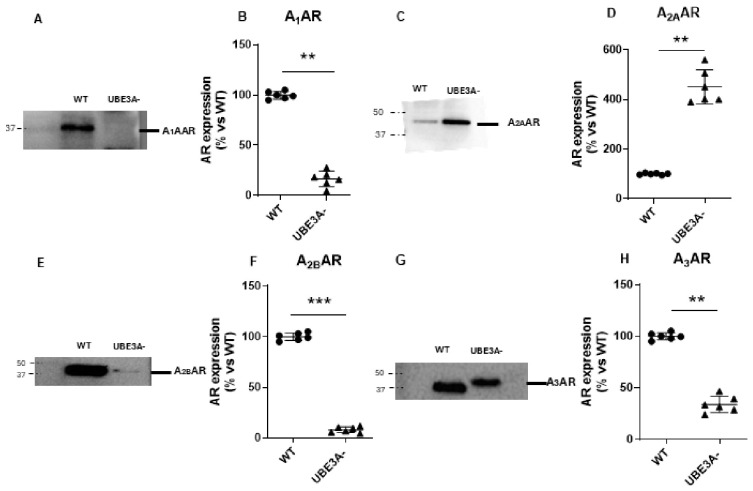
Adenosine receptor expression in WT and UBE3A^−^ SH-SY5Y cells. Cell lysates from control (WT) and UBE3A^−^ SH-SY5Y cells were used for Western blotting analysis using specific antibodies to recognize ARs. A ‘stain-free protein normalization’ method was used for the normalization of bands to total protein in blots, eliminating the need for housekeeping proteins. (**A**,**C**,**E**,**G**) Representative images of Western blot experiments; (**B**,**D**,**F**,**H**) densitometric analyses of the Western blot immunoreactive bands; closed circles represent the WT cellular model, while triangles represent the UBE3A^−^ model, data are expressed in % vs. WT control samples, and represent a semiquantitative analysis (*n* = 6, mean ± SEM). ** *p* < 0.01, *** *p* < 0.001, vs. WT, Student’s *t*-test.

Western blot analysis of SH-SY5Y lysates revealed a significant reduction in A_1_AR, A_2B_AR, and A_3_AR protein levels in UBE3A-silenced cells compared with their wild-type (WT) controls (Figure 1A,B,E–H). In contrast, A_2A_AR levels were significantly augmented in cells silenced for UBE3A^−^ (Figure 1C,D).

### 3.2. Effects of AR Stimulation on Cellular Metabolic Rate/Survival

To investigate the effects of AR stimulation on neuronal metabolic rate/survival, neuronal differentiated SH-SY5Y cells were treated with AR agonists (CHA for A_1_R, CGS or NECA for A_2A_R, BAY-606583 for A_2B_R, and Cl-IBMECA for A_3_R), at concentrations able to activate the receptors.

**Figure 2:**
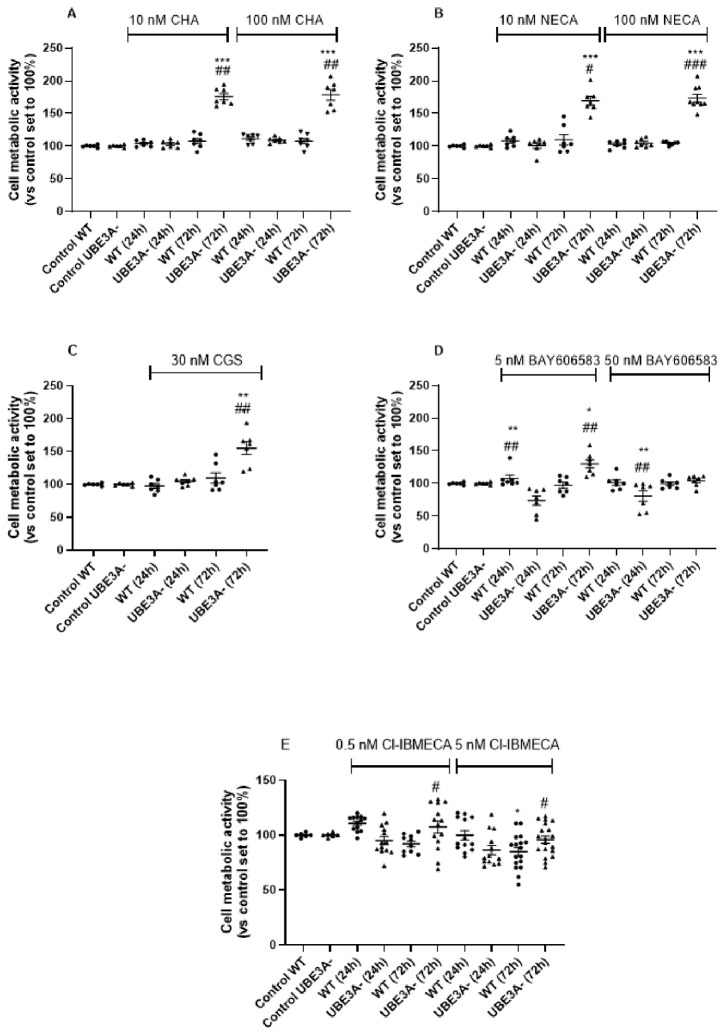
Stimulation of adenosine receptors modulates metabolic activity in SH-SY5Y cells. WT and UBE3A^−^ SH-SY5Y cells were challenged with the indicated concentrations of: A1R agonist CHA (panel **A**), A2AR agonist NECA (panel **B**), A2AR selective agonist CGS (panel **C**), A2BR agonist BAY606583 (panel **D**), or A3R agonist Cl-IBMECA (panel **E**), for 24 h or 72 h. Following treatments, MTS metabolic activity (proxy for viability) was measured. Values represent background-subtracted absorbance at 490 nm normalized to the mean of untreated control wells on the same plate (control = 100%), closed circles represent the WT cellular model, while triangles represent the UBE3A^−^ model,. Data are expressed as mean ± SEM from n ≥ 6 independent experiments, each performed in technical duplicates or triplicates. * *p* < 0.05, ** *p* < 0.01, *** *p* < 0.001, vs. control WT or UBE3A^−^ (untreated); # *p* < 0.05, ## *p* < 0.01, ### *p* < 0.001, vs. the corresponding WT or UBE3A^−^ time point. Statistical analysis: one-way ANOVA followed by Bonferroni post-test.

As shown in Figure 2A, UBE3A^−^ cell metabolic rate, measured by the mean of the MTS assay, was significantly increased after a 72 h stimulation of A_1_R with CHA, at both the concentrations tested. In contrast, no significant effect was observed on WT cells (Figure 2A). As confirmation, the selective A_1_R antagonist DPCPX abolished the CHA-induced increase in cell metabolic rate (Supplementary Figure S1A).

Similar results were obtained with the non-selective ARs agonist NECA (10 nM and 100 nM; Figure 2B), which activate A_2A_AR but also A_1_AR at the tested concentrations. To confirm the Ars’ specificity, experiments were repeated in the presence of the selective A_2A_R antagonist ZM241358: ZM reduced the NECA-induced increase in cell survival in UBE3A^−^ SH-SY5Y cells (Supplementary Figure S1B). In parallel, the selective A_2A_ agonist CGS significantly boosted the metabolic rate of UBE3A^−^ cells (Figure 2C) and, again, it had no significant effect on WT cells (Supplementary Figure S1B), confirming that A_2A_AR activation enhances the metabolic rate of UBE3A^−^ SH-SY5Y cells.

By contrast, A_2B_AR stimulation produced a time-dependent effect: exposure to the A_2B_AR agonist BAY-606583 (5–50 nM) decreased cell viability after 24 h in UBE3A^−^ cells but not in WT cells (Figure 2D). However, UBE3A^−^ cells showed a rescue in their metabolic rate/viability after 72 h of treatment with BAY-606583.

Finally, Cl-IBMECA (A_3_AR agonist, at 0.5 nM and 5 nM) did not induce changes in cell viability and metabolic rate compared to the untreated controls, in either WT or UBE3A^−^ cells (Figure 2E). Because MTS reduction reflects metabolic activity rather than absolute cell counts, these data indicate altered metabolic/viability responses to AR stimulation. Nevertheless, based on these results, the metabolic rate of UBE3A-silenced SH-SY5Y cells was modulated by the activation of both A_1_AR and A_2A_ARs, and so subsequent experiments focused on these AR subtypes.

### 3.3. cAMP Signaling

Dose–response experiments with AR agonists (CHA for A_1_R, CGS or NECA for A_2A_AR, BAY-606583 for A_2B_R, and Cl-IBMECA for A_3_AR) were performed in both WT and UBE3A^−^ cells, in order to verify their intracellular activity on cAMP levels and the presence of putative differences in ligand affinity to the respective ARs.

**Figure 3:**
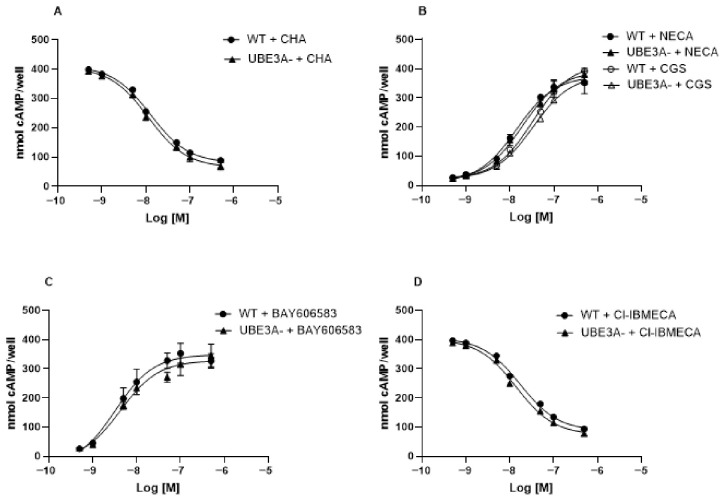
Adenosine receptor-mediated modulation of intracellular cAMP levels in WT and UBE3A-deficient cells. WT and UBE3A^−^ cells were stimulated with the indicated concentrations of adenosine receptor agonists. Specifically, cells were treated with 10 µM FK and increasing concentrations of the A1R agonist CHA (panel **A**), the A2AAR agonists NECA and CGS21680 (panel **B**), the A2BAR agonist BAY 60-6583 (panel **C**), or 10 µM FK and increasing concentrations of the A3R agonist Cl-IBMECA (panel **D**). Intracellular cAMP levels were measured and expressed as nmol cAMP per well, and concentration–response curves were generated for WT and UBE3A^−^ cells. Corresponding EC50 values are reported in the text. Data represent mean ± SEM from at least three independent experiments performed in duplicate.

CHA (Figure 3A) induced a dose-dependent inhibition of FK-induced cAMP release, with a potency (EC50) value of 13.1 ± 1.2 nM in WT cells. A comparable affinity value was evidenced for CHA in UBE3A^−^ cells (10.9 ± 1.1 nM, Figure 3A), along with a similar increased receptor efficacy in UBE3A^−^ cells with respect to WT ones (Emax: 84.9 ± 6.6 nmol cAMP/well in WT cells, 79.8 ± 1.2 nM in UB3A- cells).

NECA and CGS produced a clear dose-dependent *stimulation* of cAMP accumulation in differentiated SH-SY5Y cells (Figure 3B): EC50 was comparable between both WT and AS cells (NECA: 18.4 ± 1.7 nM in WT cells; 19.2 ± 1.8 nM in UBE3A^−^ cells; CGS: 32.7 ± 3.1 nM in WT cells; 33.4 ± 3.0 nM in UBE3A^−^ cells). The drug’s efficacy (Emax) was slightly enhanced in UBE3A^−^ cells, indicating an increased functional coupling of A2AAR to the cAMP pathway in the UBE3A-deficient background (Emax for NECA: 365 ± 26 nmol cAMP/well in WT cells, 398 ± 25 nmol cAMP/well in UB3A- cells;).

BAY606583 also evoked a dose-dependent enhancement of cAMP levels, with comparable EC50 value in WT and UBE3A^−^ cells (EC_50_= 3.6 ± 0.2 nM in WT cells; 3.9 ± 0.2 nM in UBE3A^−^ cells) (Figure 3C).

Finally, Cl-IBMECA elicited a dose-dependent inhibition of FK-induced cAMP levels, consistent with the A_3_R coupling to Gi proteins (Figure 3D). The drug’s inhibitory profile was similar in WT and UBE3A^−^ cells, and no significant differences were detected between WT and UBE3A^−^ cells across the concentration range tested (EC_50_ = 17.4 ± 1.8 nM in WT cells; 14.1 ± 1.5 nM in UBE3A^−^ cells), with similar Emax values (WT cells: 91 ± 19 nmol cAMP/well; UBE3A^−^ cells: 89 ± 23 nmol cAMP/well) (Figure 3D).

Overall, these data show that receptor functionality and ligand affinity for each AR subtype is preserved in UBE3A-deficient cells, whereas receptor efficacy, most notably for A_2A_R, is increased in the UBE3A^−^ background, consistent with the enhanced functional coupling of A_2A_R to the cAMP pathway in UBE3A-deficient cells.

### 3.4. Kinetics of Adenosine Receptor Desensitization

To investigate the AR desensitization process, we examined the functional responsiveness of ARs following repeated or prolonged agonists’ exposure. A sustained agonist stimulation usually produces GPCR desensitization, including AR desensitization [7,26], and so we investigated if the desensitization kinetics would differ between WT and UBE3A^−^ cells. To this purpose, WT and UBE3A-deficient SH-SY5Y cells were pretreated with the AR agonist (100 nM CHA or NECA) for increasing intervals (5–120 min), washed, and then challenged with a standardized secondary stimulation (10 µM forskolin + 10 nM CHA for A1R; 10 nM NECA for A2AAR) before measuring intracellular cAMP.

**Figure 4:**
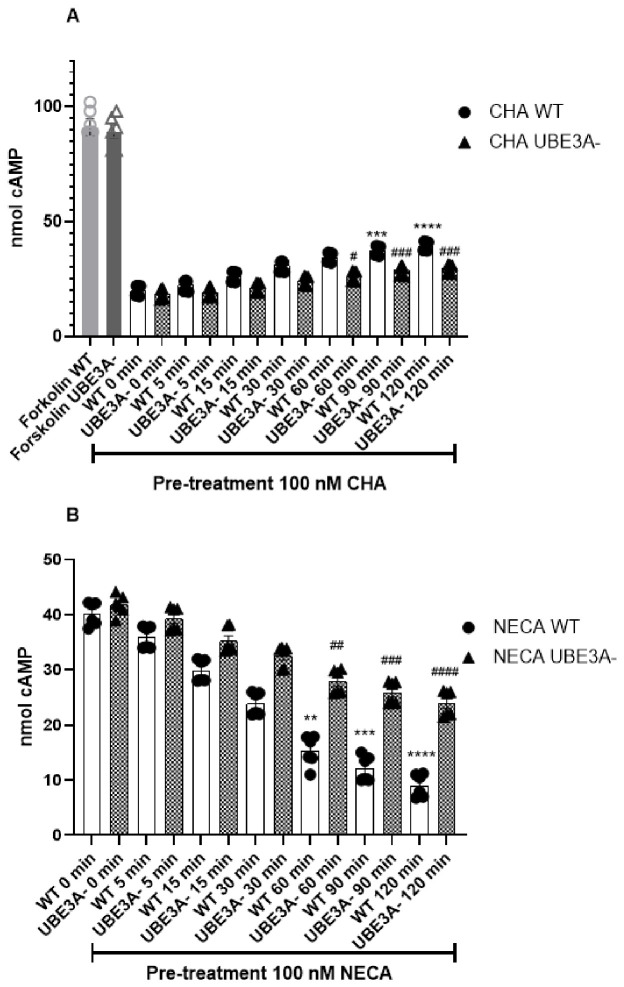
Kinetics of adenosine receptor desensitization in WT and UBE3A-deficient cells. (**A**) WT (white bars) and UBE3A^−^ (checkerboard-patterned bars) cells were pre-treated with medium alone or the A_1_R agonist CHA (100 nM) for the indicated time points (5–120 min), washed, and subsequently stimulated with forskolin (FK) and 10 nM CHA for an additional 15 min. “0 min” indicates cells not pretreated with the agonist. (**B**) WT (white bars) and UBE3A^−^ (checkerboard-patterned bars) cells were pre-treated with medium alone or the A_2A_AR agonist NECA (100 nM) for the indicated time points (5–120 min), washed, and subsequently stimulated with 10 nM NECA for an additional 15 min. “0 min” indicates cells not pretreated with the agonist, closed circles represent the WT cellular model, while triangles represent the UBE3A^−^ model, Data are expressed as mean ± SEM with the individual data points shown. Statistical significance was determined by one-way ANOVA followed by Bonferroni post-test. ** *p* < 0.01, *** *p* < 0.001, **** *p* < 0.0001 vs. WT 0 min; # *p* < 0.05, ## *p* < 0.01, ### *p* < 0.001, #### *p* < 0.0001 vs. corresponding WT time points.

CHA potently reduced the increase in cAMP levels induced by the cyclase activator FK (Figure 4A). This Gi-mediated effect was progressively lost in cells exposed to the A1AR agonist for prolonged time periods, suggesting the occurrence of A_1_AR desensitization in WT cells (white columns, Figure 4A). In UBE3A^−^ cells, the ability of CHA to block FK-mediated cAMP accumulation was maintained strongly following the exposure to the A_1_AR agonist, in particular after longer exposure times (Figure 4A). The kinetics of desensitization was significantly faster in WT versus UBE3A^−^ cells (half-time; t½ = 32.59 ± 1.92 min for WT cells; t½ = 41.52 ± 2.52 min for UBE3A^−^ cells, *p* = 0.0182, unpaired *t*-test), thus suggesting that in UBE3A-silenced neuronal cells, A_1_AR functionality is preserved longer, and A_1_AR was desensitized later.

NECA produced a robust, concentration-dependent stimulation of cAMP accumulation (Figure 3B). In WT cells, the NECA-induced cAMP response declined progressively with prolonged agonist exposure, consistent with a time-dependent desensitization of A_2A_R (white columns, Figure 4B). By contrast, UBE3A^−^ cells maintained a larger residual cAMP response after the same prolonged NECA treatments (checkerboard-patterned bars, Figure 4B). Quantitative kinetic analysis confirmed that desensitization proceeded significantly faster in WT than in UBE3A^−^ cells (t1/2 = 35.50 ± 2.87 min in WT cells for WT cells; 45.43 ± 3.05 min for UB3A- cells, *p* = 0.0392, unpaired *t*-test). These results suggest the presence of reduced A_2A_R desensitization in the UBE3A-deficient cells.

### 3.5. GRK2 Interaction with ARs and Desensitization Machinery

Because G protein-coupled receptor kinases, such as GRK2, modulate ARs’ phosphorylation and internalization, we assessed the GRK2 levels in SH-SY5Y cells after prolonged AR agonists’ exposure to test whether differences in GRK2 expression or regulation account for the preserved AR signaling in UBE3A-deficient cells [20,21].

**Figure 5:**
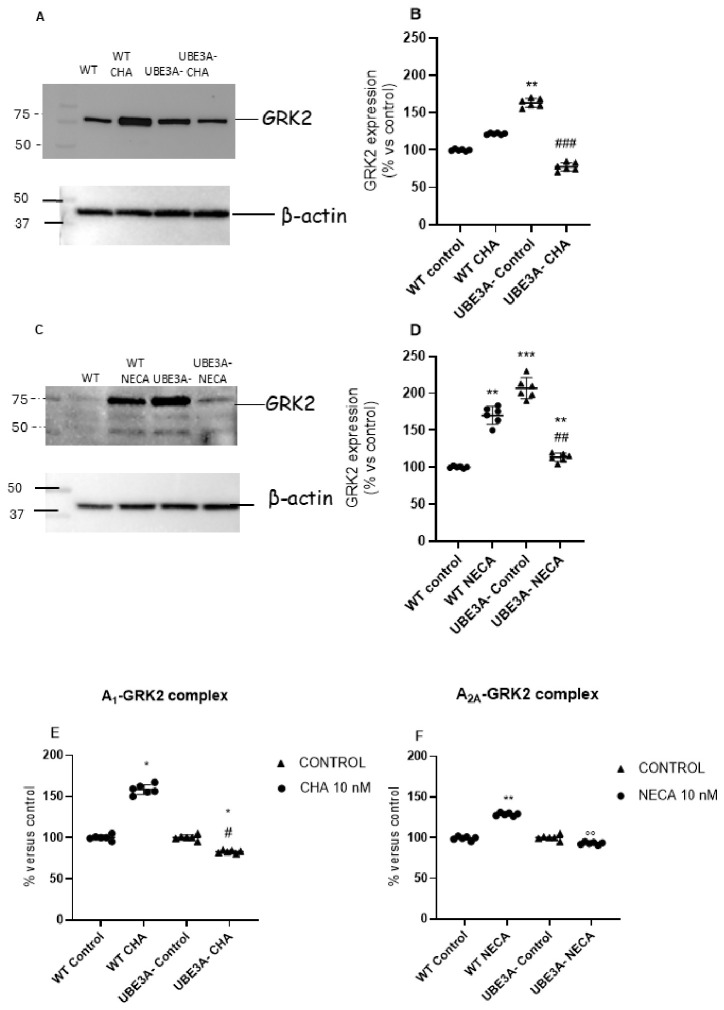
GRK2 expression and GRK2–adenosine receptor interaction in WT and UBE3A-deficient cells. WT and UBE3A^−^ cells were treated for 30 min with CHA (10 nM), NECA (10 nM), or medium alone (control). GRK2 expression was assessed by Western blot with total protein normalization. Representative blots are shown (**A**,**C**), with β-actin used as loading control, and densitometric analysis of GRK2 bands is presented (**B**,**D**) as % of control cells (mean ± SEM, *n* = 6). GRK2 association with A1AR or A2AAR was measured by immunoenzymatic assay (**E**,**F**) and expressed as % of control and WT cells (mean ± SEM, n ≥ 6), closed circles represent the WT cellular model, while triangles represent the UBE3A^−^ model,. Statistical significance was determined by one-way ANOVA followed by Bonferroni post-test. * *p* < 0.05, ** *p* < 0.01, *** *p* < 0.001, vs. WT control; # *p* < 0.05, ## *p* < 0.01, ### *p* < 0.001, vs. UBE3A^−^ control.

We focused on GRK2 because GRK2/3 are the principal cytosolic kinases recruited to activated class A GPCRs via liberated Gβγ and are widely implicated in the homologous desensitization of neurotransmitter receptors [26,27]. In basal untreated conditions, UBE3A^−^ cells presented significantly higher levels of GRK2 at the plasma membrane level with respect to WT (Figure 5A–D), with β-actin used as loading control (Figure 5A–C). In WT cells, GRK2 expression at the plasma membrane significantly increased upon stimulation with both A_1_R or A_2A_R agonists, as expected. These data are in fact consistent with the GRK recruitment necessary to initiate receptors’ desensitization after their activation. Interestingly, in UBE3A^−^ cells, upon CHA or NECA stimulation, GRK2 levels at the plasma membrane decreased (Figure 5A–D); indeed, GRK2 exhibited an opposite trend compared to WT cells.

Consistent with these data, the GRK2 association with A_1_ and A_2A_Rs increased in WT samples following 30 min of stimulation with CHA or NECA (Figure 5E,F). In UBE3A^−^ cells, GRK2–AR complex formation was not boosted by A_1_ and A_2A_ stimulation, and instead was significantly reduced, at WT basal level. These data suggest the presence of a deficit in the GRK2-dependent desensitization pathway for both A_1_ and A_2A_ ARs when UBE3A is absent in SH-SY5Y cells.

### 3.6. Proteasome System and Neurite Morphology upon AR Stimulation

Following our investigation of AR signaling and desensitization in UBE3A^−^ neuronal cells, we next turned to investigating ARs’ effects on the intracellular proteostasis machinery. Building on evidence that UBE3A loss perturbs the ubiquitin–proteasome system and autophagy pathways, we focused on the MDM2–p53 regulatory axis and autophagic flux.

**Figure 6:**
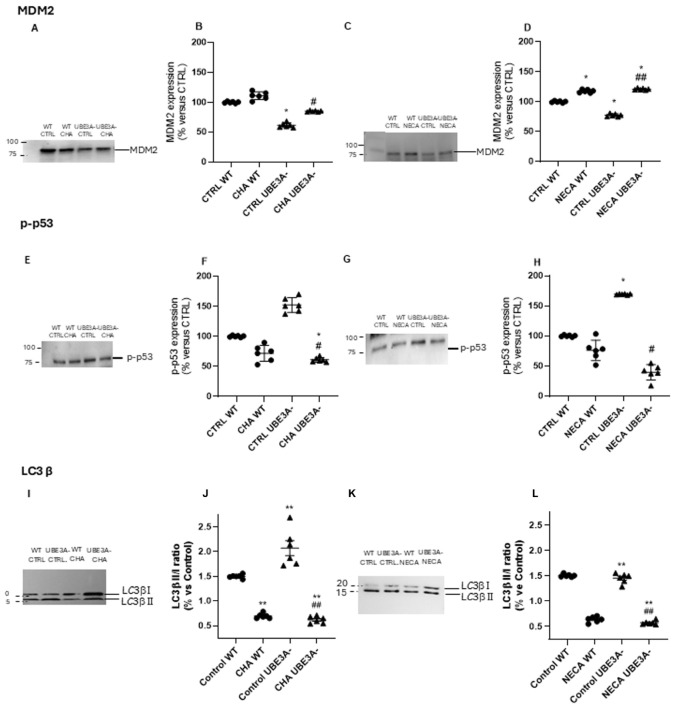
Effects of A_1_AR and A_2A_AR stimulation on MDM2-p53 axis and autophagy flux in WT and UBE3A^−^ cells. WT and UB3A- cells were challenged with medium alone (untreated control cells) or 10 nM CHA or 10 nM NECA, as indicated. At the end of treatment, cell lysates were used for Western blot analyses using an antibody specific for MDM2 (panels **A**–**D**), phospho-p53 (panels **E**–**H**), or LC3b (panels **I**–**L**). A ‘stain-free protein normalization’ method was used for the normalization of bands to total protein in blots, eliminating the need for housekeeping proteins. The data are expressed as % vs. control cells and represent a semiquantitative analysis (*n* = 6, mean ± SEM). (**A**,**C**,**E**,**G**,**I**,**K**) Representative images of Western blot analysis. (**B**,**D**,**F**,**H**,**J**,**L**) Densitometric analysis of immunoreactive bands. The data are expressed as % vs. control and WT, mean ± SEM with individual data points shown, closed circles represent the WT cellular model, while triangles represent the UBE3A^−^ model, Statistical significance was determined by two-way ANOVA, considering cell genotype (WT vs. UBE3A^−^) and treatment (control, CHA, NECA) as independent variables, followed by Bonferroni’s multiple comparisons test. * *p* < 0.05, ** *p* < 0.01, vs. WT control cell; # *p* < 0.05, ## *p* < 0.01vs. UBE3A^−^ control cell.

In basal conditions, UBE3A^−^ cells displayed significantly lower levels of the ubiquitin ligase MDM2 and increased levels of activated (phosphorylated) p53 (Figure 6A–H), as also previously shown [18]. When SH-SY5Y cells were stimulated with CHA or NECA, a significant enhancement of MDM2 levels was evidenced in both WT and UBE3A^−^ cells. Accordingly, CHA and NECA reduced the levels of phosphorylated p53 (Figure 6E–H). These effects were particularly evident in UBE3A^−^ neuronal cells: this behavior is consistent with the presence of an enhanced MDM2-mediated turnover of p53, and a shift toward pro-survival signaling in UBE3A^−^ cells under A_1_ and A_2A_AR stimulation.

Regarding the autophagy process, the basal level of autophagy induction, indicated here by the LC3B II/I ratio, was higher in UBE3A^−^ cells than in WT cells (Figure 6J). We found that CHA and NECA significantly reduced LC3-Beta activation (i.e., LC3B II/I ratio) with respect to the untreated cells, for both WT and UBE3A^−^ cells (Figure 6I–L). These effects were particularly interesting in UBE3A^−^ cells where there is an hyperactivation of LC3-mediated autophagy steps [22].

To determine whether these changes reflected alterations in autophagic flux rather than steady-state LC3 levels, additional experiments were performed in the presence or absence of bafilomycin A1 (Supplementary Figure S2). In WT cells, CHA and NECA reduced LC3-II levels under basal conditions, while co-treatment with bafilomycin A1 resulted in a marked accumulation of LC3-II compared to bafilomycin A1 alone, indicating enhanced autophagic flux.

**Figure 7:**
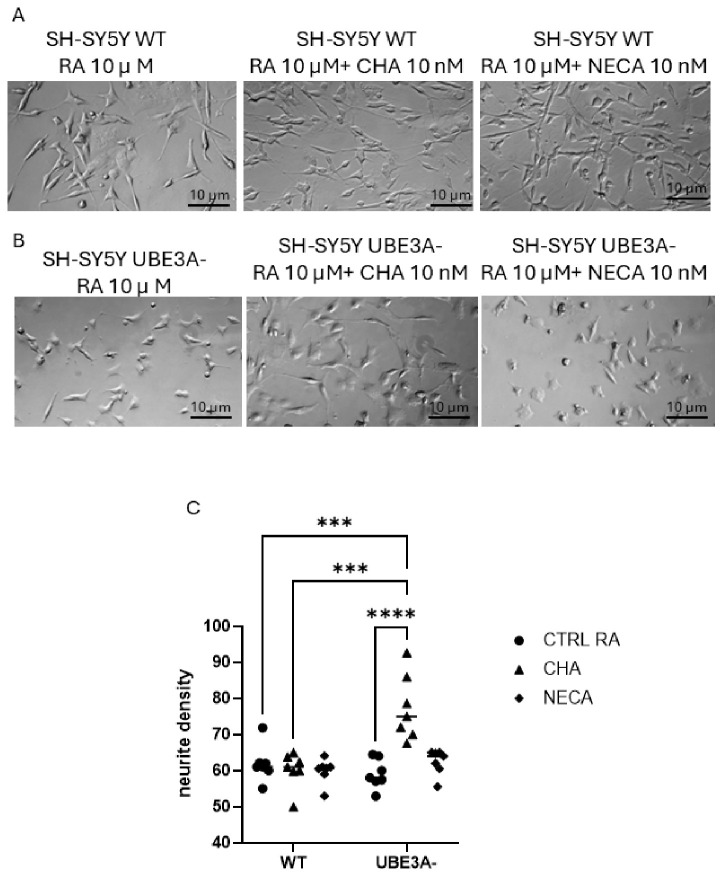
Effects of adenosine receptor agonists on neuronal morphology in SH-SY5Y cells. WT (panel **A**) and UBE3A^−^ (panel **B**) cells were treated with retinoic acid (RA, 10 µM) alone, or in combination with CHA (10 nM) or NECA (10 nM) for 4 days. (**A**,**B**) Representative phase-contrast images of SH-SY5Y WT cells differentiated with RA alone or with CHA or NECA. (**C**) Quantitative analysis of neuritic density in SH-SY5Y WT and UBE3A^−^ cells. Neuritic density was assessed using ImageJ, applying a minimum neurite length threshold of 10 µm. Neurite outgrowth was quantified as the average neurite length per cell, calculated as the total neurite length divided by the number of neurites and expressed in micrometers (µm). For each condition, two independent wells were analyzed, with three images per well (six images per condition). The number of cells per image varied; at least 20 cells per condition were included. Data are shown as individual data points with mean ± SEM. Statistical significance was determined by one-way ANOVA followed by Bonferroni post-test *** *p* < 0.001, **** *p* < 0.0001 vs. WT control cells.

To evaluate if AR signaling also influences neuronal morphological aspects, we examined the effects of ARs’ pharmacological modulation on neuronal morphology, looking at the neurites’ density. We treated WT and UBE3A^−^ cells with A_1_ and A_2A_R agonists and measured their neuritic network development. WT and UBE3A^−^ cells showed a similar neurite network development (Figure 7). The treatment with the A_1_R agonist CHA significantly enhanced neurite density only in UBE3A^−^ cells, while no significant effects on neurite density were evidenced following A_2A_R activation (Figure 7).

## 4. Discussion

Herein, we show that the loss of UBE3A in a neuronal cell model is associated with a selective remodeling of the adenosinergic receptor system, at the level of the A_1_ and A_2A_ receptors in particular, and an impaired desensitization of ARs that may involve, at least in part, altered GRK2 dynamics. The functionality of ARs is similar between WT and UBE3A^−^ cells, but A_1_R and A_2A_R are desensitized slower in UBE3A^−^ cells. A_1_R and A_2A_R agonists preferentially enhance cell metabolic rate/viability in UBE3A-deficient cells, and modulate the MDM2–p53 axis and autophagy markers. These results connect receptor-level changes to downstream proteostasis and morphological phenotypes that are relevant to AS pathophysiology.

Beyond its canonical role in ubiquitin-dependent proteostasis, UBE3A has been reported to regulate and modulate multiple signaling pathways in neurons, often essential for neuronal growth and homeostatic stability [28,29]. Among these pathways, adenosine receptors (A_1_AR, A_2A_R, A_2B_AR, A_3_AR) are key modulators of neurodevelopment, influencing neurite outgrowth, synaptogenesis and activity-dependent circuit refinement [30,31,32]. Dysregulated adenosinergic signaling has been implicated in AS-related motor and cognitive deficits and a selective A_2A_AR blockade has shown therapeutic promise in the preclinical AS murine model, thus appearing to ameliorate hippocampal-dependent learning strategies, LTD deficits, and motor impairment [5]. The adenosinergic system is a “fine modulator” of neuronal homeostasis and synaptic plasticity, often activated in noxious conditions [33]. These considerations provide a mechanistic rationale for examining deeper, at the molecular level, how UBE3A loss reshapes adenosine receptors’ expression and signaling in neuronal cells, and how such remodeling intersects with neuronal proteostasis and morphology.

Herein, protein expression data indicate a selective downregulation of A1AR, A2BAR, and A3AR in UBE3A-deficient SH-SY5Y cells, together with an upregulation of A2AR. In vivo, an increased density of A_2A_R has been reported in the hippocampus of Ube3a^m-/p+^ mice [28], but no changes have been reported in their striatum [34].

In this AS framework, A_1_ and A_2A_ receptors resulted in the most relevant receptors. In fact, their activation was in fact able to increase the metabolic/viability rate of UBE3A^−^ silenced SHSY5Y cells. This relevance could be expected, considering the prominent role of these AR subtypes in neuronal tissues [35]. Because MTS measures metabolic activity, at this moment, we cannot distinguish increased cell number from altered metabolic state or reduced apoptosis. Moreover, altered neuronal proliferation is not a well-established feature of AS in vivo. The dominant literature highlights maturation/synaptic defects in AS mouse models rather than robust proliferation changes, while human iPSC models similarly point to delayed maturation rather than clear altered proliferation [36,37]. Of note, altered neural progenitor proliferation and neurogenesis timing is a recurring feature across several NDs, including autism spectrum disorders [38,39]. Nevertheless, with the absence of in vivo data, we therefore interpret our proliferation data cautiously and propose targeted developmental assays in primary neurons and in vivo to determine whether AR modulation affects progenitor proliferation in AS models.

All ARs are functionally coupled to G proteins, and importantly, modulate cAMP levels. We performed here dose–response functional experiments with selective agonists in both WT and UB3A- cells, in order to verify putative differences in ligand affinity to the respective ARs, and thus in AR activity. Because cAMP is a principal second messenger downstream of ARs, the altered cAMP responses we observe in UBE3A-deficient cells provide a mechanistic bridge between receptor expression/desensitization changes and downstream effects, although we acknowledge that additional non-canonical pathways (for example β-arrestin signaling or localized cAMP microdomains) may also contribute. Of note, efficacy (E max) was slightly enhanced in UBE3A^−^ cells for A_2A_AR, indicating an increased functional coupling of A2AAR to the cAMP pathway. The enhanced A_2A_AR efficacy is consistent with prior reports implicating A_2A_AR overactivation in AS [5].

Our experiments confirmed the presence of a time-dependent A1AR and A2AR desensitization [40] in WT and UBE3A^−^ SH-SY5Y cells. Quantitative kinetic analysis revealed a slower desensitization of both A_1_AR and A_2A_AR in UBE3A-deficient cells with respect to WT cells, thus suggesting that UBE3A^−^ loss can cross-talk with ARs. To prolong the AR signaling in the context of UBE3A loss could have several reasons and also consequences, as both feedback or counteracting mechanisms for synaptic modulation, homeostasis, and metabolic regulation, given the broad roles of A_1_ and A_2A_ receptors in the brain [31].

Consistent with this view, we found A_1_ and A_2A_ AR activation induced a reduced GRK2 recruitment at the plasma membrane and impaired GRK2–AR complex formation after agonists’ exposure in UBE3A^−^ cells. The parallel increase in GRK2 at the plasma membrane in UBE3A^−^ cells in basal untreated conditions suggests a system ready to desensitize GPCR receptors in general, but not ARs specifically. Impaired GRK2 function is a plausible mechanism that could contribute to prolonged AR signaling and the downstream changes in proteostasis and neuritogenesis, even if this hypothesis remains to be tested directly by targeted perturbation experiments (for example, GRK2 knockdown or overexpression or selective pharmacological modulation of GRK2 activity). Of note, we focused on GRK2 because GRK2/3 are the principal cytosolic kinases recruited to activated class A GPCRs via liberated Gβγ and are widely implicated in homologous desensitization of neurotransmitter receptors; however, our data are correlative and do not exclude contributions from other GRKs or regulatory proteins [17,26,27,41].

Nevertheless, these findings support a model in which UBE3A loss disrupts the early steps of GPCRs desensitization (i.e., receptor phosphorylation by GRKs and subsequent scaffolding events), thereby prolonging receptors’ signaling or altering their temporal profile. Because GRK2 also participates in receptor trafficking and cross-talk with other signaling cascades, its dysregulation may have broad consequences for synaptic responsiveness and homeostatic adaptation. Restoring appropriate GRK2 recruitment or downstream desensitization pathways could therefore normalize receptor kinetics and downstream signaling in UBE3A-deficient neurons, as similarly demonstrated for other GPCRs in precursor cell commitment [21].

The activation of A_1_ and A_2A_ AR in UBE3A-deficient cells increases MDM2 levels and reduces phosphorylated p53 levels, consistent with an enhanced p53 turnover and a shift toward pro-survival signaling in cells. Concomitant reductions in LC3B II/I ratio indicate that A_1_ and A_2A_ AR stimulation is able to modulate the autophagic flux in UBE3A-deficient cells, where these autophagy markers are elevated in basal conditions. These findings align with the established role of MDM2 as a negative regulator of p53 and with the tight coupling between p53 activity, autophagy, and cell fate decisions in stressed or transformed cells [32]. These results connect the fine-tuning purinergic signaling to proteostasis networks already implicated in UBE3A deficiency; UBE3A loss in fact perturbs ubiquitin-dependent degradation and autophagy [22,42].

To evaluate how adenosine-receptor signaling influences neuronal cells, we examine the effects of pharmacological modulation of ARs on neurite networks. In line with previous reports [23,34], the overall neurite network is similar in WT and UBE3A^−^ SH-SY5Y cells. However, neurite density is significantly enhanced by A1AR stimulation in UBE3A^−^ cells, showing another effect of AR pathways. Consistent with our data, A1AR activation has been shown to promote neurite extension and neuronal differentiation in several in vitro systems, even if its specific effect depends on cell type, developmental stage, and signaling context [43]. The literature widely evidence how adenosine and its receptors are well described as modulators of neuronal development, but the net morphological outcome depends on the balance and temporal profile of subtype activation and synaptic plasticity [44],

Loss of UBE3A in our model is associated with a coordinated remodeling of AR expression (reduced A1, A2B, A3, and increased A2AARs) and with slower A1/A2A AR desensitization linked, at least in part, to altered GRK2 dynamics [45,46]. These receptor-level changes could prolong A1AR signaling and bias downstream pathways toward cytoskeletal remodeling and neuritogenesis. The pro-survival shifts we observed (enhanced MDM2, reduced phospho-p53, and lowered LC3B II/I after A1AR stimulation) provide a plausible molecular substrate for enhanced neurite elaboration, since reduced p53 activity and modulation of autophagy have both been linked to increased neurite outgrowth and structural plasticity in neuronal models.

Overall, our findings suggest adenosinergic signaling, in particular with A_1_ and A_2A_AR, as possible candidate therapeutic targets in AS. Pharmacological modulation of A_2A_AR has shown promise in preclinical AS models [5], but therapeutic strategies must account for the complex interplay with proteostasis pathways and the risk of maladaptive chronic signaling when desensitization machinery is impaired.

Key limitations of the present study include reliance on an immortalized neuronal line for many mechanistic assays; region- and neuron-type specificity in vivo remain to be fully defined. To strengthen translational relevance, future work should validate GRK2–AR dysregulation in primary neurons and defined brain regions across developmental stages, and test whether restoring GRK2 function (genetic rescue or pharmacologic modulation) normalizes desensitization, cAMP dynamics, and proteostasis.

## 5. Conclusions

Loss of UBE3A produces a coordinated set of alterations in adenosine receptor expression, desensitization machinery, and effects on proteostasis that together can reshape neuronal signaling and morphology. Our study provides a mechanistic framework for how altered purinergic signaling may contribute to but also counteract AS pathogenesis and highlights actionable molecular nodes for future therapeutic exploration. We acknowledge that SH-SY5Y cells are an immortalized model and that neurite density changes in this system require validation in primary neurons and in vivo. Future experiments should test whether A1AR-driven neuritogenesis in UBE3A-deficient neurons translates into functional synaptic connectivity and whether restoring GRK2 recruitment or normalizing AR desensitization prevents the morphological effect.

## Data Availability

The original contributions presented in this study are included in the article/Supplementary Material. Further inquiries can be directed to the corresponding author.

## Supplementary Materials

The following supporting information can be downloaded at: https://www.mdpi.com/article/10.3390/jdb14020020/s1, Figure S1: Specificity of AR stimulation on cell survival in SH-SY5Y cells; Figure S2: Effects of adenosine receptor stimulation on autophagic flux in WT SH-SY5Y cells; Supplementary Excel S1: raw data.

## Abbreviations

The following abbreviations are used in this manuscript:

AS	Angelman syndrome
AR	Adenosine receptor
A_1_AR	Adenosine A1 receptor
A_2A_AR	Adenosine A2A receptor
A_2B_AR	Adenosine A2B receptor
A_3_AR	Adenosine A3 receptor
UBE3A	Ubiquitin protein ligase E3A
WT	Wild type
SH-SY5Y	Human neuroblastoma SH-SY5Y cell line
cAMP	Cyclic adenosine monophosphate
GPCR	G protein-coupled receptor
GRK2	G protein-coupled receptor kinase 2
CHA	N6-cyclohexyladenosine
NECA	5′-N-ethylcarboxamidoadenosine
CGS21680	Selective A2A adenosine receptor agonist
BAY 60-6583	Selective A2B adenosine receptor agonist
Cl-IB-MECA	Chloro-IB-methyladenosine
DPCPX	8-Cyclopentyl-1,3-dipropylxanthine
ZM241385	Selective A2A adenosine receptor antagonist
