# Supplementary material for: Adenosine Receptor Functionality and Desensitization Machinery in a Neuronal Cell Model of Angelman Syndrome
Source: J Dev Biol. 2026 May 2;14(2):20. doi: 10.3390/jdb14020020 (PMC13214759; doi:10.3390/jdb14020020)
Supplement: Supplementary file 1 [file jdb-14-00020-s001.zip › Supplementary Figures.pdf]

Article

Adenosine receptor functionality and desensitization machinery in cellular model of Angelman Syndrome

Martina Contestabile[1], Jacqueline Fátima Martins de Almeida[1], Chiara De Cesari[2], Ilaria Tonazzini[2], Paolo Giovanni Artini[3*], Simona Daniele[1]

1 *Department of Pharmacy, University of Pisa, 56126 Pisa, Italy;*

2 *Istituto Nanoscienze, Consiglio Nazionale delle Ricerche (CNR), @NESTe, Piazza San Silvestro 12, 56127 Pisa, Italy;*

3 *Department of Clinical and Experimental Medicine, University of Pisa, 56126 Pisa, Italy*

* *Correspondence: P.G.A., paolo.artini@unipi.it; Tel.:+39050554104*

Supplementary Figure 1

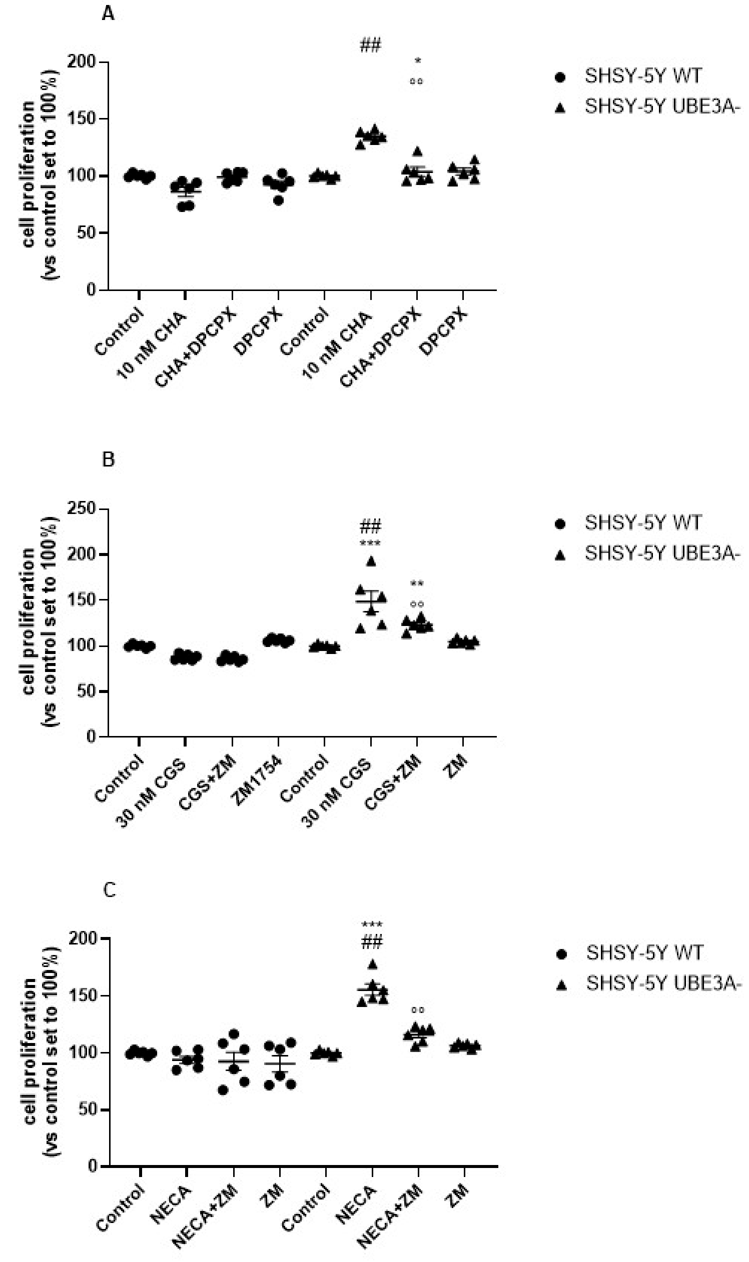

Supplementary Figure S1. Specificity of AR stimulation on cell survival in SH-SY5Y cells. WT and UB3A- SHSY-5Y cells were challenged with the indicated concentrations of the A1AR agonist CHA (panel A), A2AAR agonist CGS (panel B), A2AAR agonist NECA (panel C), in the absence or in the presence of the respective AR antagonist: DPCPX for A1AR (panel A) or MRS1754 for A2AAR (panel B and panel C)) for 72 h. Following treatment, cell survival was measured by MTS assay. The data are expressed as % versus untreated control cells. The data are expressed as % vs. control and WT cells n

≥ 6 ± SEM. Statistical significance was determined by one-way ANOVA followed by Bonferroni post-test. *P < 0.05, **P < 0.01, ***P < 0.001, ****P < 0.0001 vs. control cell; #P < 0.05, ##P < 0.01, ###P < 0.001, ####P < 0.0001 vs. WT cell; °P < 0.05, °°P < 0.01, °°°P < 0.001, °°°°P < 0.0001 vs agonist alone.

Supplementary Figure 2

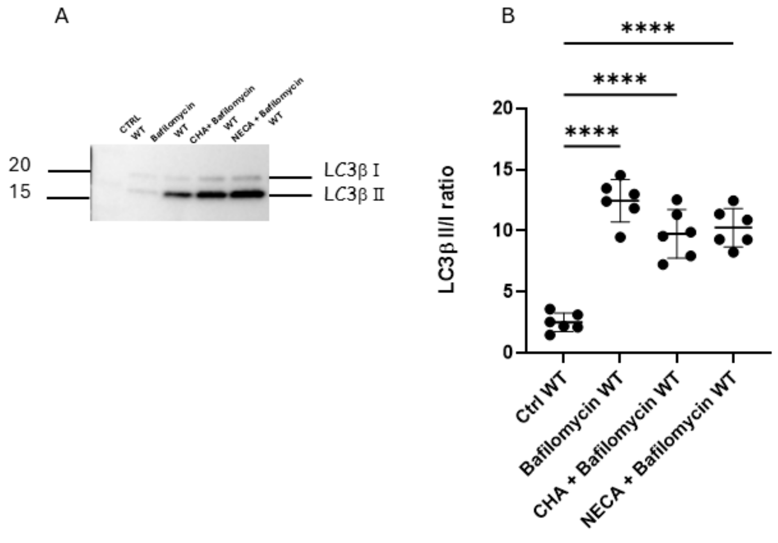

Supplementary Figure S2. Effects of adenosine receptor stimulation on autophagic flux in WT SH-SY5Y cells. WT cells were challenged with medium alone (untreated control cells), bafilomycin A1 (200 nM, 4 h), or bafilomycin A1 in combination with CHA or NECA (10 nM), as indicated. Bafilomycin A1 was added during the last 4 h of treatment following agonist exposure. At the end of treatment, cell lysates were used for western blot analyses using an antibody specific for LC3B. A 'stain-free protein normalization' method was used for the normalization of bands to total protein in blots. The data are expressed as % vs. control cells and represent a semiquantitative analysis (n ≥ 3, mean ± SEM). (A) Representative images of western blot analysis. (B) Densitometric analysis of immunoreactive bands expressed as LC3B II/I ratio, with individual data points shown. Statistical significance was determined by one-way ANOVA followed by Bonferroni post-test. *P < 0.05, **P < 0.01, ***P < 0.001, ****P < 0.0001 vs. control cells.
